# Effects of Six Sequential Charged Particle Beams on Behavioral and Cognitive Performance in B6D2F1 Female and Male Mice

**DOI:** 10.3389/fphys.2020.00959

**Published:** 2020-08-28

**Authors:** Jacob Raber, Andrea Fuentes Anaya, Eileen Ruth S. Torres, Joanne Lee, Sydney Boutros, Dmytro Grygoryev, Austin Hammer, Kristin D. Kasschau, Thomas J. Sharpton, Mitchell S. Turker, Amy Kronenberg

**Affiliations:** ^1^Department of Behavioral Neuroscience, Oregon Health & Science University, Portland, OR, United States; ^2^Departments of Neurology and Radiation Medicine, Division of Neuroscience ONPRC, Oregon Health & Science University, Portland, OR, United States; ^3^Oregon Institute of Occupational Health Sciences and Department of Molecular and Medical Genetics, Oregon Health & Science University, Portland, OR, United States; ^4^Department of Microbiology, Oregon State University, Corvallis, OR, United States; ^5^Department of Statistics, Oregon State University, Corvallis, OR, United States; ^6^Biological Systems and Engineering Division, Lawrence Berkeley National Laboratory, Berkeley, CA, United States

**Keywords:** object recognition, home cage activity, CD68, BDNF, gut microbiome, charged particle radiation, galactic cosmic radiation, helium ions

## Abstract

The radiation environment astronauts are exposed to in deep space includes galactic cosmic radiation (GCR) with different proportions of all naturally occurring ions. To assist NASA with assessment of risk to the brain following exposure to a mixture of ions broadly representative of the GCR, we assessed the behavioral and cognitive performance of female and male C57BL/6J × DBA2/J F1 (B6D2F1) mice two months following rapidly delivered, sequential 6 beam irradiation with protons (1 GeV, LET = 0.24 keV, 50%), ^4^He ions (250 MeV/n, LET = 1.6 keV/μm, 20%), ^16^O ions (250 MeV/n, LET = 25 keV/μm 7.5%), ^28^Si ions (263 MeV/n, LET = 78 keV/μm, 7.5%), ^48^Ti ions (1 GeV/n, LET = 107 keV/μm, 7.5%), and ^56^Fe ions (1 GeV/n, LET = 151 keV/μm, 7.5%) at 0, 25, 50, or 200 cGy) at 4–6 months of age. When the activity over 3 days of open field habituation was analyzed in female mice, those irradiated with 50 cGy moved less and spent less time in the center than sham-irradiated mice. Sham-irradiated female mice and those irradiated with 25 cGy showed object recognition. However, female mice exposed to 50 or 200 cGy did not show object recognition. When fear memory was assessed in passive avoidance tests, sham-irradiated mice and mice irradiated with 25 cGy showed memory retention while mice exposed to 50 or 200 cGy did not. The effects of radiation passive avoidance memory retention were not sex-dependent. There was no effect of radiation on depressive-like behavior in the forced swim test. There was a trend toward an effect of radiation on BDNF levels in the cortex of males, but not for females, with higher levels in male mice irradiated with 50 cGy than sham-irradiated. Finally, sequential 6-ion irradiation impacted the composition of the gut microbiome in a sex-dependent fashion. Taxa were uncovered whose relative abundance in the gut was associated with the radiation dose received. Thus, exposure to sequential six-beam irradiation significantly affects behavioral and cognitive performance and the gut microbiome.

## Introduction

Space radiation poses a hazard to flight crews during potential deep space missions or prolonged stays on the moon. NASA has concerns about potential impacts of such exposures on behavior, cognition, and performance of astronauts. The galactic cosmic radiation (GCR) includes all naturally occurring ions from protons to uranium present in differing proportions and across a wide range of energies (NCRP 132). Prior NASA-supported behavioral and cognitive studies have typically involved single particle exposures with protons (Rabin et al., 1991; Bellone et al., 2014; Sweet et al., 2014; Impey et al., 2016), ^16^O (Sweet et al., 2014; Raber et al., 2015b; Rabin et al., 2015), ^56^Fe (Kandasamy et al., 1994; Rabin et al., 2000; Cherry et al., 2012), ^28^Si (Bellone et al., 2014; Raber et al., 2014, 2015b), or combined effects of two particle exposures, such as protons (150 MeV/n, 0.1 Gy) and ^56^Fe ions (600 MeV/n, 0.5 Gy; Raber et al., 2015a), on hippocampal function. There is a clear gap, however, in our knowledge regarding exposure to complex radiation fields involving a more diverse grouping of charged particles, a scenario very pertinent to exposures of astronauts during space missions.

Recently, we reported the effect of sequential irradiation with three charged particles: protons (1 GeV, 60%), ^16^O (250 MeV/n, 20%), and ^28^Si (263 MeV/n, 20%) at 0, 25, 50, or 200 cGy. Both male and female mice were irradiated at 4–6 months of age. The effects of this 3-ion exposure were assessed on behavioral and cognitive performance, cortical levels of brain-derived neurotrophic factor (BDNF), CD68 (macrosialin), Microtubule associated protein 2 (MAP-2), and the gut microbiome (Raber et al., 2019). When circadian activity levels were assessed, male mice irradiated with 50 cGy showed higher activity levels in the home cage during the dark (active) period than sham-irradiated mice. Mice irradiated with 50 cGy also showed increased depressive-like behavior in the forced swim test. Sham-irradiated mice and mice irradiated with 25 cGy showed object recognition, but mice irradiated with 50 or 200 cGy did not. In females, but not males, there were increased CD68 levels following irradiation. In males, but not females, there were reduced BDNF levels following irradiation. A significant positive correlation between BDNF and CD68 levels was observed, suggesting a role for activated microglia in the alterations in BDNF levels. Finally, sequential three-beam irradiation impacted the diversity and composition of the gut microbiome, with dose-dependent impacts and alterations to the relative abundance of several gut genera, such as *Butyricicoccus* and *Lachnospiraceae*.

The release of BDNF is important for cognitive performance. Activation of microglia is known to trigger the release of BDNF, which in turn induces the prolonged activation of microglia (Gomes et al., 2013; Mizobuchi et al., 2014; Zhang et al., 2014). CD68 (macrosialin), a lysosome-associated membrane glycoprotein, is a marker of activated microglia (Neuen-Jacob et al., 1993; Tanaka et al., 2013). An increase in activated microglia is seen in the medial prefrontal cortex of 6-month-old male transgenic Thy1-EGFP MJrsJ mice at 15 and 20 weeks following ^16^O (600 MeV/n; 5, or 30 cGy) or ^48^Ti (600 MeV/n; 5, or 30 cGy) ion irradiation (Parihar et al., 2016).

Microtubule associated protein 2 is a dendritic protein important for stabilizing microtubuli and dendritic plasticity, required for dendrite elongation (Harada et al., 2002). MAP-2 is a sensitive marker for age-related changes in rodents (Benice et al., 2006; Peister et al., 2006) and non-human primates (Haley et al., 2010) and can be affected by irradiation (Villasana et al., 2010a; Olsen et al., 2014). Therefore, we also assessed MAP-2 levels in the current study.

The effects of space radiation on the brain might involve alteration in the diversity of the gut microbiome and gut-liver-brain axis (Tilg and Kaser, 2011; Foster and McVey Neufeld, 2013; Ohland et al., 2013; Ritchie et al., 2015; Ghaisas et al., 2016; Allen et al., 2017; Casero et al., 2017; Turroni et al., 2017) reviewed in Cervantes and Hong (2016). Prior work has demonstrated that the mouse gut microbiome is impacted by 13 days of space flight (Ritchie et al., 2015). Subsequent investigations have sought to determine the factors that could contribute to space flight induced changes in the microbiome, finding that the mouse gut microbiome is sensitive to ^16^O ion irradiation (600 MeV/n, 0.1 and 0.25 Gy; Casero et al., 2017) and sequential irradiation with graded doses of protons (1 GeV, 60%), ^16^O (250 MeV/n, 20%), and ^28^Si (263 MeV/n, 20%; Raber et al., 2018). Moreover, radiation-based alterations to the gut microbiome may help explain some of the observed behavioral and cognitive impacts of radiation, given the mounting research that points to the microbiome’s influence on the brain and behavior (Cryan and O’Mahony, 2011; Cryan et al., 2020).

In the present study, we assessed the effects of six rapidly delivered sequential ion beams on behavior and cognitive performance of female and male B6D2 F1 mice to more closely approximate the diversity of ions and LET’s in deep space. We queried whether the observed effects are associated with alterations in cortical and hippocampal BDNF, MAP-2, and CD68 levels. In addition, we examined charged particle-induced changes in the diversity and composition of the gut microbiome to explore potential changes to the gut-brain axis.

## Materials and Methods

### Animals, Radiation Exposure, Study Design, and Home Cage Monitoring

Animal care and irradiation exposures were performed as described in our previous study (Raber et al., 2019). A limitation of translational interpretation of rodent central nervous system (CNS) space radiation studies is that many are pursued using C57BL/6 inbred mice. Mouse mutants generated on different genetic backgrounds revealed the effect of the genetic background on modulating the phenotypes seen (Sigmund, 2000). Therefore, to generate converging evidence it is important to assess effects of space radiation in mice on other genetic backgrounds as well. F1 hybrid mice display hybrid vigor, with increased disease resistance, better survival under stress, greater natural longevity, and larger litters. Because of the hybrid vigor and genetic and phenotypic uniformity, F1 hybrid mice are often preferred over outbred mice in radiation research (Lutz et al., 2012). C57BL/6J (B6) × DBA2/J F1 (D2) mice are suitable for CNS radiation studies as they are able to perform hippocampus-dependent cognitive tests well (Crawley, 2000). Briefly, F1 generation male and female mice were bred by crossing C57BL/6J (B6) and DBA2/J F1 (D2) mice. Following up on our previous three sequential beam study, involving graded doses of protons (1 GeV, 60%, LET = 0.24 keV/μm), ^16^O ions (250 MeV/n, 20%, LET = 25 keV/μm), and ^28^Si ions (263 MeV/n, 20%, LET = 78 keV/μm; Raber et al., 2018), the current six sequential beam study was pursued over the same dose range. A total of *N* = 99 mice, 4–6 months of age, were sham-irradiated or received the following exposures in rapid sequence, with three mice irradiated at the same time at the NASA Space Radiation Laboratory (NSRL) at Brookhaven National Laboratory (BNL): protons (1 GeV, LET = 0.24 keV/μm, 50% of the total dose), ^4^He ions (250 MeV/n, LET = 1.6 keV/μm, LET = 1.6 keV/μm, 20% of the total dose), ^16^O ions (250 MeV/n, LET = 25 keV/μm, 7.5% of the total dose), ^28^Si ions (263 MeV/n, LET = 78 keV/μm, 7.5% of the total dose), ^48^Ti ions (1 GeV/n, LET = 107 keV/μm, 7.5% of the total dose), and ^56^Fe ions (1 GeV/n, LET = 151 keV/μm, 7.5% of the total dose). Group sizes are as follows: *N* = 99 mice, 51 females (sham irradiation: *n* = 12 mice, 25 cGy: *n* = 15 mice, 50 cGy: *n* = 12 mice, and 200 cGy: *n* = 12 mice) and 48 males (sham irradiation: *n* = 12 mice, 25 cGy: *n* = 12 mice, 50 cGy: *n* = 12 mice, and 200 cGy: *n* = 12 mice). Doses were chosen based on our prior work with individual charged particle beams. The lower total doses are relevant to planned exploration class missions, including travel to Mars (Hassler et al., 2014). The highest dose was included as an “anchor” point, as in our previous three-beam study (Raber et al., 2018). All beams were delivered at high dose-rates (>40 cGy/min) and the time needed to switch from one beam to the next was on the order of 1–2 min. Both irradiated and sham-irradiated mice were brought to the NSRL on the exposure day and were loaded into Plexiglas holders drilled with multiple air holes. Sham-irradiated mice were loaded into holders but were not placed on the beam line. All irradiations were completed in 1 day.

Dosimetry was performed with a series of parallel plate ionization chambers calibrated with a NIST-traceable thimble ionization chamber (EG&G) according to our standard methods (Kronenberg et al., 2009). One week after sham-irradiation or exposure, the mice were returned to OHSU for behavioral and cognitive testing starting two months following exposure.

Mice were group housed at OHSU. Mice were housed at three mice per cage on a ventilated Thoren rack throughout the experimental period, except during 1 week when a subset of 24 female mice (week 1) or 24 male mice (week 2), (*n* = 6 mice/dose/sex) were singly housed and home cage activity was monitored on a conventional Metro rack using an MLog (BioBServe, Germany) home cage activity system, as described (Johnson et al., 2015). All mice were kept under a constant 12-h light: 12-h dark cycle, and water and food (PicoLab Rodent Diet 20, no. 5053; PMI Nutrition International, St. Louis, MO, United States) were provided *ad libitum.* Mice were behaviorally and cognitively tested during the light period. All procedures were approved by the Institutional Animal Care and Use Committees at OHSU and BNL and were in compliance with all federal regulations.

### Behavioral and Cognitive Testing

All behavioral and cognitive testing was conducted at OHSU by experimenters who were blinded to radiation dose. Home cage activity testing began 2 months after the irradiations and tests were conducted over a period of two weeks. Body weights were recorded prior to assessment of home cage activity. Starting on day 1 of week 3, the week following the last week of home cage activity testing, exploratory activity and measures of anxiety were assessed in the open field for 3 days. The mice were tested for novel object recognition during the two subsequent days. Starting on day 1 of week 4, depressive behavior was assessed using the forced swim test. During the remainder of week 4, hippocampus-dependent contextual, and hippocampus-independent cued fear learning and memory was assessed. During week 5, fear learning and memory were assessed using the passive avoidance test. The behavioral and cognitive paradigms used are described in detail below.

### Open Field and Novel Object Recognition

The open field was used to assess measures of anxiety, locomotor, and exploratory behavior. Mice were singly placed in a brightly lit (average: 400 lux) clear plexiglass arena (40.64 cm × 40.64 cm; Kinder Scientific, Poway, CA, United States) for five-min trials, once each day for three consecutive days. Arenas were cleaned with 0.5% acetic acid between each trial. Two white noise generating devices, (average: 85 dB, Kinder Scientific, Poway, CA, United States) were used, one on each side of the arena platforms. Movement of the mice and durations spent in the center of the arena (center 20 cm square area) were recorded and analyzed using Ethovision XT 7 video tracking software (Noldus Information Technologies, Wageningen, Netherlands). After habituation to the arena for 3 days, novel object recognition was assessed by placing two identical objects 10 cm apart in the arena on the fourth day, then replacing one object with a novel object on the fifth day. Trials on the fourth and fifth days were 10 min each. Videos recorded were viewed by experimenters who hand-scored time spent exploring each object. Time spent exploring the novel object versus the familiar object on day 5, expressed as a percentage of the total object exploration time in the trial, was used to determine object recognition memory.

### Porsolt Forced Swim Test

The Porsolt forced swim test (Porsolt et al., 1977) was used to assess depression-like behavior. Each mouse was placed individually in a 2000 mL glass beaker (diameter = 12.7 cm) containing 1600 mL of room temperature (21°C) water for a single six-min trial (4 glass beakers were used per trial to test 4 mice simultaneously). Videos were recorded and the last 5 min of each trial hand-scored for time spent immobile. Immobility was characterized by cessation of any movement other than the minimum motion needed to keep the head above water. The percentage of time spent immobile was used as a measure of depression-like behavior and/or learned helplessness.

### Fear Conditioning

Contextual and cued fear conditioning were used to assess hippocampus-dependent contextual associative memory and hippocampus-independent cued associative memory (Anagnostaras et al., 2010) using near-infrared (NIR) video and Video Freeze automated scoring software (Med Associates Inc., St. Albans, VT, United States). In the fear conditioning tests, mice learn to associate an environmental context and cue (tone, conditioned stimulus, CS) with a mild foot shock (unconditioned stimulus, US). Upon re-exposure to the training context, or a new environment in which the mice are exposed to a tone that was present during training, associative learning is assessed based on freezing behavior, characterized by absence of all movement besides respiration. On the first day (training), the mice were individually placed inside a white LED lit (100 lux) fear conditioning chamber with a metal grid floor and allowed to habituate for a 90 s baseline period. This was followed by an 80 dB, 2800 Hz tone (CS or cue) lasting for 30 s and co-terminating with a 2 s, 0.7 mA foot shock (unconditioned stimulus or US) at 120 s. Five tone-shock pairings were used, with an inter-stimulus interval (ISI) of 90 s. Measurements of average motion (cm/s) and percentage of time freezing were analyzed during the baseline period (prior to the first tone), and during each subsequent ISI and CS (tone/cue) to assess acquisition of fear memory. Chambers were cleaned between trials with a 0.5% acetic acid solution. On day two, the mice were placed back into the same context as used on the training day for a single five-min trial, and freezing behavior was measured in the absence of either tones or shocks to assess contextual associative memory. 4 h later, the mice were placed into a novel context, containing a smooth white plastic covering the wire grid floor, a “tented” black plastic ceiling, and scented with hidden vanilla extract-soaked nestlets. The chambers were cleaned between trials with a 10% isopropanol solution. Each trial consisted of a 90-s baseline, followed by a 180-s 80 dB, 2800 Hz tone. Freezing behavior was analyzed as an indicator of cued associative memory.

### Passive Avoidance

Emotional learning and memory were assessed using the passive avoidance test. Mice were placed individually into a passive avoidance chamber, consisting of two sound-attenuating, equally sized compartments (24.5 cm × 19 cm × 23 cm) separated by a gate (Hamilton-Kinder, Poway, CA, United States). On the first day (training), after a 5-s habituation, a light turned on in the compartment containing the mouse. Simultaneously, the gate to the adjoining dark compartment opened. Mice prefer to cross into the dark compartment rather than remaining in brightly lit environments. After crossing over into the dark compartment, the mice received a 3-s foot shock (0.35 mA), and the mice were immediately removed from the chamber. Mice that did not cross over into the dark compartment within 60 s were removed from the analysis (1 sham-irradiated mouse, 3 mice irradiated with 25 cGy, 1 mouse irradiated with 50 cGy, and 3 mice irradiated with 200 cGy). Mice were removed immediately from the chamber after crossing over. Latency to cross on day 2, up to a maximum of 300 s, was recorded. No shock was administered during the day 2 trials. On both days, chambers were cleaned between trials with 0.5% acetic acid.

### MAP-2, CD68, and BDNF ELISAs

For assessments of cortical MAP-2, CD68, and BDNF levels, the mice were euthanized by cervical dislocation without anesthesia and hippocampal and cortical regions of their brains dissected for separate analyses. Tissue collection occurred about 3 months following sham-irradiation or exposure to the six rapidly switched sequential ion beams at BNL. The hippocampi and cortex of each mouse brain were homogenized and a protein assay was performed using a BCA kit (Fisher Scientific, Chicago, IL, United States), as described. MyBioSource CD68 (Catalog number MBS2601301) and MAP-2 (Catalog number MBS725632, San Diego, CA, United States) ELISAs and a VWR mouse BDNF (Catalog number 10205-700, Radnor, PA, United States) ELISA were used to determine hippocampal and cortical levels of CD68, MAP-2, and BDNF according to the manufacturer instructions. Days post-irradiation was counterbalanced for samples used in these ELISAs as two mice per day were dissected for an independent project involving cultured cells. The standard curve was run in duplicate and the samples as single samples. Based on the optical density values read using a SpectraMax iD5 (Molecular Devices, San Jose, CA, United States), the MAP-2, CD68, and BDNF levels in the samples were calculated using GraphPad Prism software (San Diego, CA, United States).

### Microbiome Sequencing

Fecal boli samples for each mouse were collected during open field testing (2 months post-irradiation) and stored at −80°C until analyses could be performed. Bacterial 16S rDNA was extracted from fecal boli samples and 16S rDNA sequences were amplified and sequenced as previously described (Gaulke et al., 2016) and in a manner consistent with our prior study on mixed-beam irradiation (Raber et al., 2018). Briefly, DNA was extracted from collected fecal pellets using the Qiagen DNeasy PowerSoil Kit (Qiagen, Hilden, Germany) and the V4 region of the 16S rDNA gene was amplified in triplicate using the Earth Microbiome Project 16S PCR protocol. PCR reactions were cleaned with the UltraClean PCR clean-up kit (Qiagen, Hilden, Germany), quantified on a Qubit using the dsDNA HS Assay kit, and diluted to produce 200 ng of DNA per sample. The prepared libraries were submitted to the Oregon State University Center for Genome Research and Biocomputing for 300 bp paired-end sequencing on an Illumina MiSeq instrument. Quality control, exact sequence variants clustering, and chimera removal were conducted using the DADA2 package (Callahan et al., 2016) for R (R Core Team 2017) as per our prior study. DADA2 also assigned taxonomy to the sequence variants utilizing the Silva taxonomic training data formatted for dada2. Sequences were rarefied to 16,513 sequences per sample.

### Statistical Analyses

All data are shown as mean ± standard error of the mean (SEM). Statistical analyses were performed using SPSS (Chicago, IL, United States). Since our previous study revealed significant sex differences (Raber et al., 2019), data of males and females were analyzed separately. Sexes were combined in certain analyses noted below when sex was found to not be a significant factor. The data were analyzed using ANOVAs with radiation as a between-group factor. Performance over multiple trials was analyzed by repeated-measures ANOVA. If violation of sphericity occurred indicating that the variances of the differences between all combinations of the groups were not equal (Mauchly’s test), Greenhouse–Geisser corrections were used. When appropriate, *post hoc* tests were used as indicated. Passive avoidance data were analyzed with paired Wilcoxon tests between the latency on days 1 and 2 for each group as the data were not normally distributed. All figures were generated using GraphPad Prism software (San Diego, CA, United States). We considered *p* < 0.05 as statistically significant. Statistical analysis of microbiome data relied on non-parametric tests of association as defined in the results.

A variety of statistical tests were applied to measure the microbiome’s association with study covariates. Kendall’s tau was used to quantify the relationship between measures of either radiation dose or behavior and measures of community biodiversity (e.g., Richness, Shannon entropy, and Simpson’s index). Non-metric multidimensional scaling based on the Bray–Curtis distance visualized the variation in microbiome community composition across samples. PERMANOVA tests, as implemented by the adonis function [R:vegan:adonis()], determined if community composition varied in association with study and individual covariates (e.g., sex, cage, radiation dose, and behavioral measures). The optimal model of association between community composition and these covariates was identified using the ordistep function [R:vegan:ordistep()] which compares step-wise iterations of models using the Akaike Information Criterion. Tests of beta-dispersion were implemented using the betadisper function [R:vegan:betadisper()]. Negative binomial regression as implemented through DESeq2 (Love et al., 2014) quantified the association between the relative abundance of specific ASVs and genera of gut microbes and covariates including radiation dose and measures of behavior. Because our prior work observed non-linear and non-monotonic associations between taxon relative abundance and study covariates (Raber et al., 2018), we binned categorical data prior to running DeSeq2, which had the effect of resolving taxa whose relative abundance significantly differed in at least one category relative to the others. For tests of association with radiation dose, we categorized samples by dose. For tests of association with behavioral covariates, we categorized samples by quartiling the distribution of the covariate in question. Multiple tests were corrected through quantification of the false discovery rate and statistically significant tests were those that returned a false discovery rate less than 0.01.

## Results

### Body Weights

**FIGURE 1:**
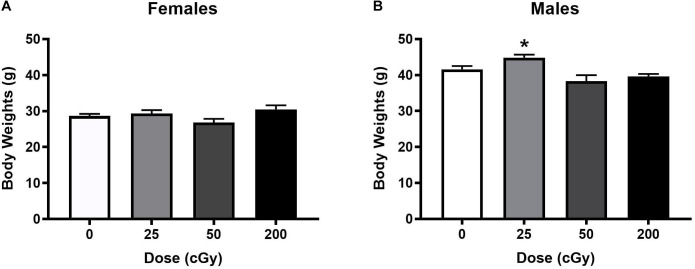
Body weights of female **(A)** and male **(B)** mice that were sham-irradiated or received six sequential beams. **(A)** There was no effect of irradiation in females. **(B)** In males, the body weights were higher in mice irradiated with 25 cGy than those irradiated with 50 or 200 cGy. *p = 0.001 versus 50 cGy and *p = 0.01 versus 200 cGy.

Based on observations by the researchers and animal staff, the rapid, sequential, six beam exposures were well tolerated by the animals. No obvious adverse effects were observed during the post-irradiation follow-up and testing periods at any dose tested. Body weights were recorded two months post-irradiation and prior to behavioral testing. There was a trend toward an effect of radiation in female mice [*F*(3,46) = 2.318, *p* = 0.088, and Figure 1A]; a significant effect of radiation in male mice [*F*(3,44) = 6.685, *p* = 0.001, and Figure 1B] was found, driven by the higher body weights in male mice irradiated with 25 cGy than those irradiated with 50 (*p* = 0.001, Sidak’s) or 200 cGy (*p* = 0.01, Sidak’s). The other individual doses could not be distinguished from the sham-irradiated control values.

### Home Cage Activity

Home cage activity of mice (*n* = 24 mice/sex, 6 mice/dose) was analyzed. There were no effects of radiation during the light or dark periods in either sex (Supplementary Figure S1).

### Activity Levels and Measures of Anxiety in the Open Field

**FIGURE 2:**
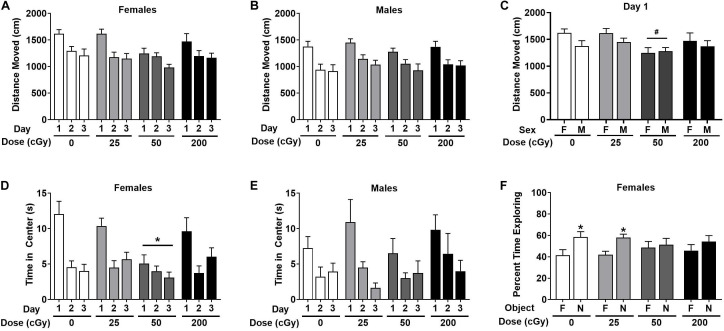
Performance of irradiated and sham-irradiated female and male mice. **(A,B)** There was no effect of radiation on activity levels over the three days of open field habituation. **(C)** On day 1 of the open field, there was a trend toward lower activity in mice irradiation with 50 cGy than sham-irradiated mice ^#^*p* = 0.058 versus 0 cGy. **(D,E)** Time female **(D)** and male **(E)** mice spent in the center of the open field. In female mice, mice irradiated with 50 cGy spent less time in the center than sex-matched sham-irradiated mice. *p = 0.014 versus 0 cGy, Dunnett’s. **(F)** Sham-irradiated female mice and female mice irradiated with 25 cGy showed object recognition but female mice irradiated with 50 or 200 cGy showed impaired object recognition and no preference for exploring the novel object. *p < 0.05 versus familiar object.

When the activity over the three days of open field habituation were analyzed in female mice, there was no effect of radiation [*F*(3,44) = 1.56, *p* = 0.21], and only an effect of day [*F*(1.74,79.99) = 28.74, *p* < 0.001, Figure 2A]. Males also demonstrated no overall effect of radiation [*F*(3,44) = 0.59, *p* = 0.63] and only an effect of day [*F*(1.51,66.44) = 52.50; *p* < 0.001, Figure 2B]. On day 1 of the open field, there was an overall effect of radiation when both sexes were analyzed together on activity levels in the open field [*F*(3,90) = 3.078, *p* = 0.031] with a trend toward lower activity in mice irradiated with 50 cGy than in sham-irradiated mice (*p* = 0.058, Dunnett’s, Figure 2C). This was not significant between sexes [*F*(1,90) = 3.040, *p* = 0.085].

Next, time spent in the more anxiety-provoking center of the open field was analyzed. In female mice, there was an effect of radiation [*F*(3,39) = 3.77, *p* = 0.018] with mice irradiated with 50 cGy spending less time in the center than sex-matched sham-irradiated mice (*p* = 0.049, Dunnett’s, Figure 2D). There was also an effect of day [*F*(2,78) = 28.11, *p* < 0.0001] with mice spending less time in the center on subsequent days, and a radiation *x* day interaction [*F*(6,78) = 2.86, *p* = 0.014]. In contrast, there was no overall effect of radiation in males [*F*(3,32) = 0.31, *p* = 0.82] and only an effect of day [*F*(2,64) = 14.52, *p* < 0.001, Figure 2E]. A similar pattern was seen when frequency to enter the center was analyzed (not shown). In females, there was a dose *x* day interaction [*F*(6,92) = 2.95; *p* = 0.011] with mice irradiated with 50 cGy entering the center less than sham-irradiated mice (sham average: 6.58 ± 0.76, 50 cGy average: 3.83 ± 0.73, *p* = 0.027, Dunnett’s) and an effect of day [*F*(2, 92) = 33.22, *p* < 0.001]. In males, there was only an effect of day [*F*(1.74, 76.56) = 23.22, *p* < 0.001] with mice entering the center less on subsequent days.

### Time Spent Exploring Objects and Novel Object Recognition

As the time spent exploring the objects and the preferential exploration of the objects in the object recognition test are distinct performance measures, they were analyzed separately. In female mice, there was no effect of radiation or day on total time spent exploring the objects when all dose groups were analyzed together (Supplementary Figure S2). In contrast, a different pattern was seen when object recognition was analyzed as performance measure. Sham-irradiated female mice showed object recognition and spent more time exploring the novel than familiar object (*t* = 3.032, *p* = 0.014, paired *t*-test, Figure 2F). Female mice irradiated with 25 cGy also showed object recognition and spent more time exploring the novel than familiar object (*t* = 2.49, *p* = 0.026). Of importance, female mice irradiated with either of the higher doses (50 or 200 cGy) showed no preference for exploring the novel object.

In male mice, there was a trend toward an effect of radiation on the total time exploring both objects over the 2 days [*F*(3,44) = 2.20, *p* = 0.10], primarily due to mice irradiated with 50 cGy exploring less than sham-irradiated mice (*p* = 0.091, Dunnett’s, Supplementary Figure S2). There was also an effect of day [*F*(1,44) = 13.52, *p* < 0.001] with mice exploring the objects more on day 2 than day 1. Sham-irradiated male mice failed to show object recognition (not shown). Male mice irradiated with 25 cGy also failed to show object recognition but male mice irradiated with 50 cGy showed object recognition (*t* = 2.41, *p* = 0.035) or 200 (*t* = 3.52, *p* = 0.0048, not shown).

### Forced Swim Test

Depressive-like behavior was assessed in the Forced Swim Test. There was no effect of radiation on percent time immobilization in female or male mice (Supplementary Figure S3).

### Fear Conditioning

Emotional learning and memory were assessed in the contextual and cued fear conditioning tests. There was no effect of irradiation on baseline motion during the 90-s baseline period (data not shown). There was also no effect of radiation on freezing during the five tones or the four ISIs (Supplementary Figure S4), supporting similar fear learning in all the groups.

When fear memory was assessed, there was no effect of radiation on freezing during the contextual nor during the cued fear memory tests (Supplementary Figure S5).

### Passive Avoidance

**FIGURE 3:**
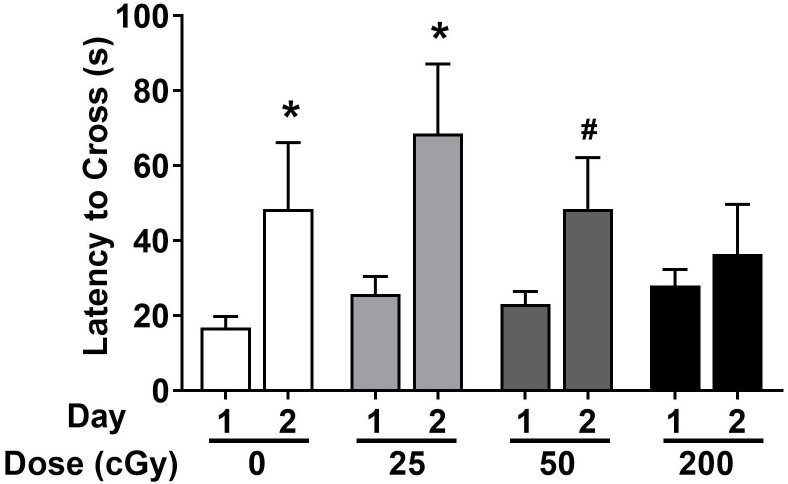
Passive avoidance learning and memory of sham-irradiated and irradiated mice. Sham-irradiated mice and mice irradiated test showed passive avoidance memory retention and entered the dark compartment later on day 2 than day 1. There was a trend toward significance in mice irradiated with 50 cGy. (*p < 0.05 versus day 1, Wilcoxon test. ^#^*p* = 0.098, Wilcoxon test).

Emotional learning and memory were also assessed in the passive avoidance test. Female and male data were combined in the analysis. In sham-irradiated mice (*p* = 0.032) and mice irradiated with 25 cGy (*p* = 0.023), latency was significantly higher on day 2 than day 1, indicating memory retention (Figure 3). While there is a trend toward a higher latency on day 2 than day 1 in mice irradiated with 50 cGy (*p* = 0.098, Figure 3), the lack of statistical significance suggests a deficit in passive avoidance memory retention. At the highest dose of 200 cGy, irradiated mice showed no passive avoidance memory retention (*p* = 0.63, Figure 3).

### Cortical and Hippocampal BDNF, CD68, and MAP-2 Levels

**FIGURE 4:**
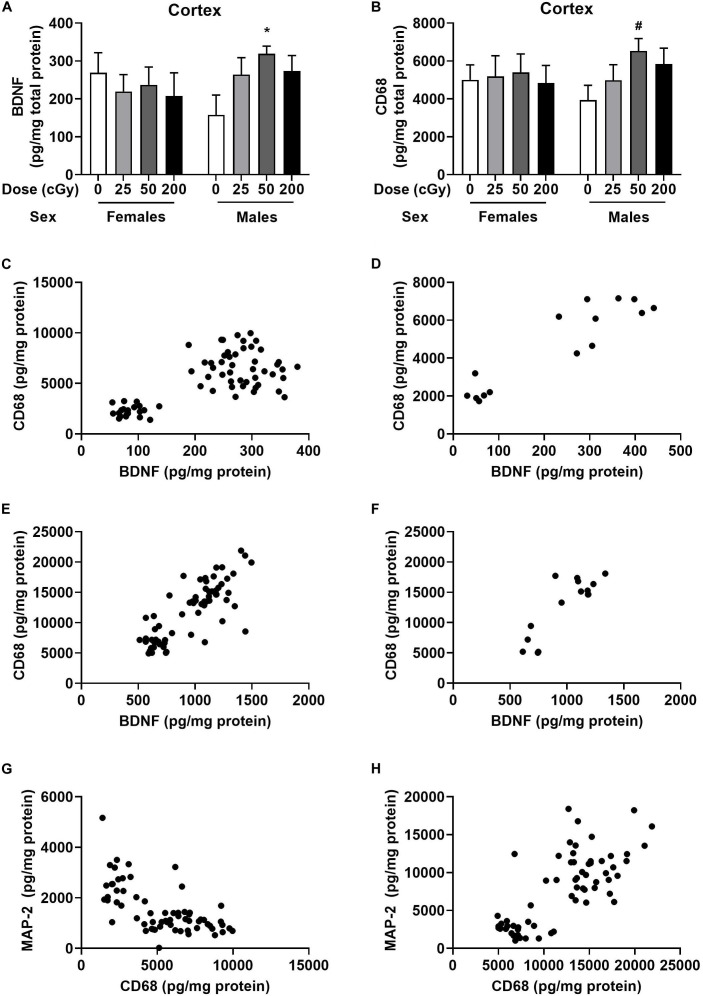
Cortical and hippocampal BDNF, CD68, and MAP-2 levels. **(A)** In males, BDNF levels in the cortex were higher levels in mice irradiated with 50 cGy than sham-irradiated mice. *p = 0.026. **(B)** In males, there was a trend toward higher CD68 levels in mice irradiated with 50 cGy than sham-irradiated mice. ^#^*p* = 0.082, Dunnett’s. **(C)** There was a positive correlation between BDNF and CD68 levels in the cortex. *r* = 0.703, *p* < 0.0001, Pearson correlation (sham-irradiated and irradiated mice combined in the analysis). **(D)** There was also a positive correlation between cortical BDNF and CD68 levels in only sham-irradiated mice. *r* = 0.907, *p* < 0.0001, Pearson correlation. **(E)** There was also a positive relationship between hippocampal BDNF and CD68 levels. *r* = 0.7985, *p* < 0.0001, Pearson correlation (sham-irradiated and irradiated mice combined in the analysis). **(F)** There was a positive correlation between hippocampal BDNF and CD68 in only sham-irradiated mice. *r* = 0.837, *p* < 0.0001, Pearson correlation. **(G)** There was a negative correlation between cortical MAP-2 and CD68 levels. *r* = −0.6314, *p* < 0.0001, Pearson correlation (sham-irradiated and irradiated mice combined in the analysis). **(H)** In contrast to the cortex, there was a positive relationship between MAP-2 and CD68 in the hippocampus. *r* = 0.7529, *p* < 0.0001, Pearson correlation (sham-irradiated and irradiated mice combined in the analysis).

Cortical and hippocampal tissues of the mice were used for analyses of BDNF, CD68, and MAP-2 levels. There was no effect of radiation on BDNF levels in cortex of females (Figure 4A). However, there was a trend toward an effect of radiation on BDNF levels in the cortex of males [*F*(3,28), *p* = 0.073], with higher levels in mice irradiated with 50 cGy than sham-irradiated mice (*p* = 0.026, Dunnett’s, Figure 4A). The CD68 levels in the cortex of females were comparable in all groups (Figure 4B). In males, there was no overall effect of radiation [*F*(3,28) = 2.86, *p* = 0.16], but the pattern was similar to that seen for BDNF, with a trend toward higher levels in mice irradiated with 50 cGy than sham-irradiated mice (*p* = 0.082, Dunnett’s, Figure 4B). There was no effect of radiation on BDNF or CD68 levels in the hippocampus of females and males, nor on MAP-2 levels in the cortex or hippocampus of females and males (Supplementary Figure S6).

We also analyzed the relationship between BDNF and CD68 levels in individual mice. There was a significant positive correlation between BDNF and CD68 levels in the cortex when the female and male data were analyzed together (Figure 4C, *r* = 0.703, *p* < 0.0001, Pearson correlation, 2-tailed, and 66 XY pairs) and when the female and male data were analyzed separately (females: *r* = 0.750, *p* < 0.0001, Pearson correlation, 2-tailed, 34 XY pairs; males: *r* = 0.627, *p* = 0.0001, Pearson correlation, 2-tailed, 32 XY pairs, data not shown). The significant positive correlation between BDNF and CD68 levels was also seen when the data from the mice that received sham-irradiation were removed from the analysis (*r* = 0.6479, *p* < 0.0001, Pearson correlation, 2-tailed, and 51 XY pairs). Of note, this relationship did not depend on radiation exposure, as a relationship between cortical BDNF and CD68 levels was also seen in sham-irradiated B6D2F1 mice (*r* = 0.907, *p* < 0.0001, Pearson correlation, 2-tailed, 15 XY pairs, Figure 4D). Similar findings were obtained in the hippocampus, where there was also a positive relationship between BDNF and CD68 levels (*r* = 0.799, *p* < 0.0001, Pearson correlation, 2-tailed, 67 XY pairs, Figure 4E). This relationship was also seen when the sham-irradiated mice were removed from the analysis (*r* = 0.787, *p* < 0.0001, Pearson correlation, 2-tailed, 51 XY pairs, data not shown) and also seen when only the sham-irradiated mice were analyzed (*r* = 0.854, *p* < 0.0001, Pearson correlation, 2-tailed, 15 XY pairs, Figure 4F). However, the relationship between BCNF and CD68 in hippocampus also did not depend on radiation exposure.

We also assessed the relationship between MAP-2 and CD68 in the cortex. There was a negative correlation between MAP-2 and CD68 (*r* = −0.6314, *p* < 0.0001, Pearson correlation, 2-tailed, 65 XY pairs, Figure 4G). The same negative correlation was seen when the female and male data were analyzed separately (females: *r* = −0.6553, *p* < 0.0001, Pearson correlation, 2-tailed, and 34 XY pairs; males: *r* = −0.5297, *p* = 0.0001, Pearson correlation, 2-tailed, 31 XY pairs, data not shown). This negative correlation remained when data of sham-irradiated mice were removed (*r* = −0.658, *p* < 0.0001, Pearson correlation, 2-tailed, 51 XY pairs, data not shown). In contrast to the cortex, there was a positive relationship between MAP-2 and CD68 in the hippocampus (*r* = 0.753, *p* < 0.0001, Pearson correlation, 2-tailed, 66 XY pairs, Figure 4H). This was also seen when only the sham-irradiated mice were analyzed (*r* = 0.796, *p* = 0.0002, Pearson correlation, 2-tailed, 16 XY pairs, data not shown). No effect of radiation dose was seen on the relationship between MAP-2 and CD68 in cortex.

**FIGURE 5:**
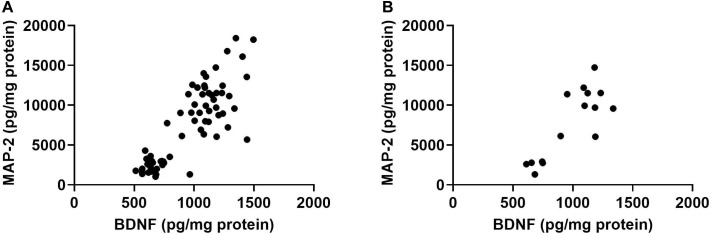
**(A)** There was a positive relationship between hippocampal BDNF and MAP-2 in the hippocampus. *r* = 0.8315, *p* < 0.0001, Pearson correlation (sham-irradiated and irradiated mice combined in the analysis). **(B)** There was a positive relationship between hippocampal BDNF and MAP-2 levels in only sham-irradiated mice. *r* = 0.8542, *p* < 0.0001, Pearson correlation.

Finally, there was a positive relationship between BDNF and MAP-2 in the hippocampus (*r* = 0.8315, *p* < 0.0001, Pearson correlation, 2-tailed, 67 XY pairs, Figure 5A). This was also seen when only irradiated (*r* = 0.8240, *p* < 0.0001, 2-tailed, and 51 XY pairs) or only sham-irradiated mice were analyzed (*r* = 0.8542, *p* < 0.0001, Pearson correlation, 2-tailed, 16 XY pairs, Figure 5B). This relationship was not seen in the cortex.

### Microbiome

**FIGURE 6:**
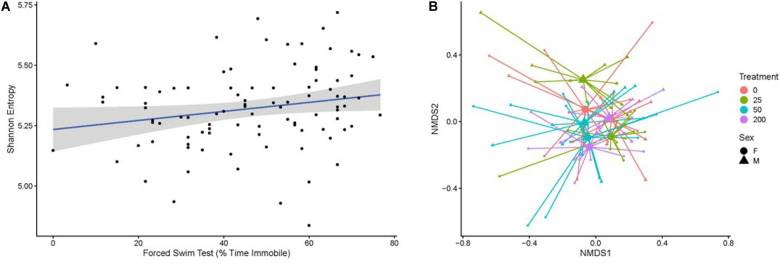
**(A)** The Shannon entropy of the gut microbiome, which is a biodiversity measure that integrates community richness and evenness, correlates with an individual’s forced swim test score (percent time immobile). Individual samples are represented by black points. The blue line indicates the best fit of the points as determined by a linear model, where the gray shading represents the 95% confidence interval of the model. **(B)** The composition of the gut microbiome relates to the amount of radiation a mouse received in a sex-dependent way, as illustrated in this non-metric multidimensional scaling plot based on the Bray–Curtis dissimilarity of the samples. Small points represent individual microbiome samples and are colored by the amount of radiation the host received. Points are connected via line segments to triangles (male mice) or circles (female mice) that represent the centroids of each sex-by-treatment cohort of mice. Permanova tests demonstrate that the microbiome significantly varies across treatment groups in a sex-dependent manner.

Fecal boli were used to profile the taxonomic composition of the gut microbiome through 16S rRNA gene sequencing. After rarefaction, a total of 4,004 taxa were identified across the mice subjected to our analysis. Gut microbiome biodiversity (i.e., alpha-diversity), whether quantified as community richness or by using measures that combine community richness and evenness (e.g., Shannon entropy, Simpson’s diversity index), did not significantly vary as a function of radiation exposure or radiation dose. However, particular measures of behavior performance correlated with the Shannon entropy or Simpson’s diversity of the gut microbiome as determined by a Kendall’s tau test (Figure 6). Specifically, these measures of diversity correlated with forced swim test immobility (Shannon *p* = 0.01692; Simpson’s *p* = 0.0211, Figure 6A) and the difference between the latency to cross on training and test days in the passive avoidance test (Shannon *p* = 0.02013; Simpson’s *p* = 0.01727).

The overall composition of the gut microbiome was significantly but weakly associated with radiation dose as measured by the Bray–Curtis dissimilarity metric (*R*^2^ = 0.04571, *p* = 0.0002). We considered that other study or individual covariates could impact the microbiome’s association with radiation exposure and applied a step-wise model selection procedure to resolve study and individual covariates that optimally explain the variation in the gut microbiome. The optimal model produced through this approach identified a significant and much stronger association between microbiome composition and the statistical interaction of the covariates sex and radiation dose (*R*^2^ = 0.11287, *p* = 0.0002). This result indicates that radiation dose has a strong impact on the composition of the gut microbiome, but that this impact differs between males and females (Figure 6B). No behavioral covariates were significantly associated with the gut microbiome composition.

We next identified specific taxa whose relative abundance in the gut associates with radiation dose to better understand the taxonomic basis underlying the above observations. Multivariate modeling of sparse taxa is typically underpowered, so we prioritized the analysis of relatively common taxa by removing ASVs that were present in fewer than 10% of samples from the analysis. We identified 37 ASVs that significantly associated with radiation dose, of which 11 showed a negative Log2 fold change and 26 showed a positive Log2 fold change. An independent analysis also resolved 11 ASVs and 3 genera that significantly differed in relative abundance as a function of the sex of the mouse. Given these observations, we then modeled ASV relative abundance as a function of the interaction between sex and radiation dose, finding 48 ASVs that vary in association with radiation in a sex-dependent manner. The results of these analyses are summarized in Supplementary Table S1.

The differential relative abundance of particular taxa also associated with scores of several behavioral performance covariates. For example, the relative abundance of 12 ASVs varied in association with latency to cross in the passive avoidance test, while 16 ASVs varied in association with the difference between the latency to cross on training and test days. Moreover, the total distance moved in the open field on day 1 associated with the relative abundance of 19 different ASVs, and total time spent in the center of the open field on day 1 linked to 15 ASVs. Finally, 24 ASVs varied with measures of novel object preference, and 15 ASVs similarly varied with the percentage of time a mouse spent immobile during the forced swim test. To obtain insight into whether the observed associations between these ASVs and the various behavioral and cognitive performance covariates are sensitive to radiation dose, we conducted a follow-up analysis using DESeq2 that quantified whether the ASV relative abundance associated with the statistical interaction between a given behavioral or cognitive performance covariate and radiation dosage. This analysis resulted in very few associations between ASV relative abundance and this statistical interaction term, possibly due to limitations in analytical power. That said, six ASVs [ASV66 (*Bacteroides*), ASV185 (Lachnospiraceae_NK4A136), ASV349 (*Bifidobacterium*); ASV469 (*Lachnospiraceae*); ASV494 (*Mucispirillum*); ASV506 (*Alistipes*); and Supplementary Table S1] linked to latency to cross in the passive avoidance test in a dose-dependent manner (fdr < 0.01). Similarly, two ASVs [ASV223 (*Parasutterella*), ASV391 (Muribaculaceae); Supplementary Table S1] linked to the latency to cross on training and test days in the passive avoidance test in a radiation dose dependent manner (fdr < 0.01). These results point to radiation dose-dependent associations between specific microbiota and behavior and cognitive performance in the mice interrogated in the present study.

## Discussion

This study shows that a six rapidly delivered, sequential charged particle beam exposure, relevant to human exposures on prolonged space missions outside the protection of the Earth’s magnetic field, has some detrimental effects on the mouse brain. Female mice irradiated with 50 cGy spent less time in the center than sham-irradiated mice in the open field. In addition, female mice irradiated with 50 or 200 cGy showed impaired object recognition, while female mice irradiated with 25 cGy were not affected. These effects were sex-dependent and were not seen in males. However, we recognize that there were sex differences in the activity levels and measures of anxiety of sham-irradiated mice in the open field and we cannot exclude the possibility that they might have contributed to sex-dependent changes in the object recognition test.

The sex of an animal has a profound effect on brain function under physiological and pathological conditions and on susceptibility and severity of various neurological conditions. There are sex differences in the regulation of immune function (Ngo et al., 2014; Klein and Flanagan, 2016) and in cardiovascular function (Denton and Baylis, 2007; Kollar and Ostadal, 2013; Wheatly et al., 2014). The results of the current study indicate that a exposure to a complex mixture of charged particles relevant to space flight has a strong impact on the composition of the gut microbiome that differs between males and females. Immune function, cardiovascular function, and the gut microbiome can all affect brain function. Female astronauts and cosmonauts have participated in space missions since 1963. Therefore, it is important to consider sex as a biological variable (Bale and Epperson, 2017; Miller et al., 2017) in assessing CNS risk of space irradiation.

Most radiation studies have examined males while a limited number of radiation studies have involved only female animals (Acevedo et al., 2008; Dayger et al., 2011; Rabin et al., 2013) or both male and female animals (Villasana et al., 2008, 2010b, 2011; Rabin et al., 2010; Haley et al., 2012; Liu et al., 2017, 2019; Kronenberg et al., 2018; Krukowski et al., 2018; Hinkle et al., 2019). In comparing the direction of sex-differences in relative susceptibility to develop radiation-induced cognitive injury, the pattern might depend on the radiation exposure. ^56^Fe ion irradiation reduces pathology in female but not male APP/PS1dE9 Tg mice. Similarly, male mice are more susceptible to radiation-induced changes in activity levels than female mice following whole body ^56^Fe ion irradiation (Liu et al., 2019). Further, male, but not female, C57BL/6J mice, show long-lasting behavioral alterations and cognitive injury following simulated GCR simulation (Krukowski et al., 2018). Male, but not female, mice on a C57BL/6J background also show alterations in spine density and morphology and increases in markers of microglia activation (Hinkle et al., 2019). On the other hand, female, but not male, wild-type mice were impaired in contextual fear conditioning following cranial ^56^Fe ion irradiation (Villasana et al., 2010b). Similarly, female, but not male, human apoE3 mice showed long-term cognitive injury following cranial ^56^Fe ion irradiation (Villasana et al., 2011). Finally, both female and male C57BL/6J mice showed cognitive injury two weeks following ^56^Fe ion irradiation (Haley et al., 2012).

The sex differences in CNS radiation response are not limited to space-relevant radiation. Females are more susceptible than males to behavioral alterations and cognitive injury following cranial gamma radiation of the developing brain (Roughton et al., 2012). In addition, female, but not male, human apoE3 and apoE4 mice were affected 3 months following cranial 137Cs irradiation (Villasana et al., 2006). Taken together, these data highlight the importance of including both sexes in radiation studies.

When emotional memory was assessed in the passive avoidance tests, mice irradiated with 50 or 200 cGy showed impaired memory retention while memory retention in mice irradiated with 25 cGy were not affected. These effects of radiation on passive avoidance memory retention were seen in both sexes. Further, BDNF levels in the cortex were higher levels in male mice irradiated with 50 cGy than sham-irradiated animals. Finally, rapid, sequential six-beam irradiation impacted the composition of the gut microbiome in a dose- and sex-dependent fashion. We note, however, that there was no apparent linkage between the levels of BDNF or CD68 and radiation dose, nor was there a link between the levels of BDNF or CD68 on behavior or performance in the present study.

The dose-response relationship seen for object recognition with impaired performance in female mice exposed to 50 or 200 cGy is particularly notable, as the identical dose-response relationship with impaired performance in female mice exposed to 50 or 200 cGy was seen following sequential three beam irradiation (Raber et al., 2019; Table 1). However, in contrast to object recognition, there were several differences comparing radiation effects on behavioral and cognitive performance in other tests following sequential three and six beam exposures (Table 1). Body weights were increased in males following 25 cGy radiation in the six-beam, but not the three-beam, study. In contrast, home cage activity in mice irradiated with 50 cGy was observed in the three-beam but not six beam study. In the open field, opposing effects of radiation, at different doses, were seen in the three- and six-beam studies; while mice irradiated with 25 cGy showed increased activity in the three-beam study, mice irradiated with 50 cGy showed decreased activity in the six-beam study. In addition, higher measures of anxiety in the open field were seen in female mice irradiated in the six-beam study, while no effects of exposure were seen on measures of anxiety in the three-beam study.

**TABLE 1:** Comparison of effects of three versus six beams in B6D2F1 mice.

Outcome measure	Three beams	Six beams
Body weight	No radiation effect	No effect of radiation
Home cage activity	Higher in 50 cGy than 0 cGy males	No effect of radiation
Open field: activity and measures of anxiety	Higher activity in 25 cGy than 0 Gy mice (both sexes).	No effect of radiation
Object recognition	Impaired in 50 and 200 cGy males and females	Impaired in 50 and 200 cGy females
Forced swim test: depressive-like behavior	Increased immobility in 50 cGy mice (both sexes)	No effect of radiation
Fear conditioning	No effect of radiation	No effect of radiation
Passive avoidance	No effect of radiation	Impaired memory retention in 200 cGy mice (both sexes)
Cortical BDNF levels	Lower in 200 cGy than 0 Gy males	
Cortical CD68 levels	Higher in 200 cGy than 0 Gy females	
Cortical CD68-BDNF relationship	Positive correlation (both sexes)	Positive correlation (both sexes)
Cortical CD68-MAP2 relationship	Negative correlation (both sexes)	Negative correlation (both sexes)
Gut microbiome	Increased alpha-diversity in irradiated male mice.	No correlation between irradiation and alpha-diversity. Microbiome composition varied in association with radiation dose in sex-dependent way
	Microbiome composition varied in association with radiation dose.	

While increased depressive-like behavior was seen in mice exposed to 50 cGy in the three-beam study, no effects of exposure were seen on depressive-like behavior in the six-beam study. Similarly, no effects of irradiation on cued fear memory were seen in either study. Finally, impaired passive avoidance memory retention in mice irradiated with 200 cGy was observed in the present six-beam study, but not earlier three-beam study. These data indicate that the object recognition test might be particularly suitable to help predict effects on cognitive performance after complex exposures to charged particle beams representative of components of the GCR. Furthermore, the object recognition test was shown to be particle sensitive for the detection of effects of space radiation in a recent review by the National Council on Radiation Protection and Measurements (Braby et al., 2019).

A positive relationship between cortical CD68 and BDNF levels in both sexes was seen in both the three and six beam studies (Table 1). This positive relationship was seen when sham-irradiated and irradiated mice were analyzed separately as well. CD68 is a marker of activated microglia, which in turn is important in neuroinflammation, and activated microglia release BDNF which in turn results in proliferation and prolonged activation of microglia (Neuen-Jacob et al., 1993; Tanaka et al., 2013). BDNF induces and is induced by nuclear factor κB (NFκB), which explains the role of BDNF in neuroinflammation. The release of BDNF is also important for cognitive performance (Lindholm et al., 1990; Wardle and Poo, 2003; Piepmeier and Etnier, 2015), neuroplasticity, and survival signaling. Consistent with this notion, inhibited BDNF transcription has been implicated in radiation-induced cognitive injury following clinically relevant doses (Ji et al., 2014; Son et al., 2015), and heterozygous BDNF knockout mice show impairments in fear extinction learning in adulthood (Psotta et al., 2013), and age-dependent impairments in conditioned fear learning (Endres and Lessmann, 2012). In addition, BDNF overexpressing male mice show improved learning and memory in the water maze and fear conditioning tests (Kopponen et al., 2004). However, these beneficial effects might be sex-dependent. BDNF overexpressing female mice show impaired working memory in a T-maze test involving hidden food pellets and pre-pulse inhibition (Papaleo et al., 2011). These data indicate that the cognitive effects of BDNF on the brain are complex and that elevated BDNF levels might be associated with both beneficial and detrimental effects. We note, however, that there was no apparent linkage between the levels of BDNF or CD68 and radiation dose, nor was there a link between the levels of BDNF or CD68 on behavior or performance in the present study.

Our analysis of the gut microbiome found that its composition, but not diversity, was sensitive to the amount of radiation a mouse received. However, this sensitivity to radiation appears to be largely sex-dependent, as male mice manifest a different pattern of response to radiation relative to female mice in terms of the types and relative abundances of taxa that comprise the gut microbiome. Many of the ASVs that differentially associate with radiation are members of the *Turicibacter* genus, which has previously been observed to increase with high doses of radiation in mice (Gerassy-Vainberg et al., 2017), though ASVs from the genus *Helicobacter* (ASV419, ASV410) and *Alistipes* (ASV231) also manifest sex-independent associations with radiation. However, many other ASVs only link to radiation dose in one of the two sexes. For example, two ASVs in the genus *Alistipes* (ASV191, ASV 283) elicit associations with radiation dose in male, but not female, mice. Our results showing sex differences in microbiome composition following six sequential particle beam exposures build upon prior work that points to sex differences in microbiome composition (Org et al., 2016). It is noteworthy that some ASVs in *Alistipes* show evidence of such a sex-dependency while others do not. Prior work finds that closely related taxa can manifest distinct, and in some cases even opposite, patterns of association with host physiology (Conley et al., 2016). Our results extend this observation by uncovering closely related taxa that appear to differentially associate with mouse sex.

Our analyses also identified associations between the gut microbiome and measures of mouse behavioral and cognitive performance. For example, the biodiversity of the gut microbiome (i.e., alpha-diversity) correlates with percent immobility in the forced swim test, a measure of depressive-like behavior, and the difference between the latency to cross on training and test days in the passive avoidance learning and memory test. While the taxonomic composition of the gut microbiome (i.e., beta-diversity) did not associate with behavioral or cognitive performance, a diverse set of ASVs linked to a wide array of behavioral and cognitive measures. For example, an ASV that could only be annotated as being a member of the family *Prevotellaceae* (ASV323) displayed a strong, negative association with the percent time a mouse spent exploring a novel object, which is an indicator of objective recognition memory. This association could result from an impact of cognition on the gut microbiome or the result of a cryptic confounding factor (e.g., changes in cognition that impact feeding rate to subsequently impact the ASV’s abundance). However, it is tempting to speculate that this association emerges because this ASV impacts the memory of the mouse. Little research has specifically considered the link between *Prevotellaceae* and behavioral or cognitive performance. The work that has been conducted to date suggests that members of this family link to behavioral and cognitive performance (Proctor et al., 2016), but that different genera within this family adopt inverse associations with measures of behavioral and cognitive performance (e.g., *Paraprevotella* inversely associates with depression while *Prevotella* positively associates with depression (Cenit et al., 2017). This observation underscores the importance of adopting techniques that provide more precise inferences of taxonomy than are often available through 16S rRNA gene amplicon sequence investigations (e.g., shotgun metagenomics; Sharpton, 2014). Moreover, we identify a multitude of ASVs within the family *Muribaculaceae*, also known as S24-7 (Lagkouvardos et al., 2019), that associate with a variety of behavioral measures. This family is both abundant and prevalent in the mouse gut, but remains poorly understood given that researchers only recently successfully cultured or sequenced genomes from strains within this family (Lagkouvardos et al., 2019). Our observations underscore the need to improve the functional characterization of this family, especially as it relates to mouse behavior and cognition.

The results of the present study are only somewhat consistent with our prior analysis of male mice subjected to sequential three particle beam irradiation. In that prior study, we found that (a) Shannon entropy linked to radiation dose, which differs from our present work, (b) radiation dose impacted the composition of the gut microbiome of male mice, which is consistent with the sex-dependent impact of radiation on microbiome composition observed here, and (c) specific taxa associate with radiation dose, though not behavioral or cognitive performance. While it is possible that differences in the type of radiation drive the variation in results between these investigations, it seems more probable that study effects confound the comparison of these investigations. A growing number of investigations have demonstrated that study effects can contribute substantial variation in microbiome diversity and composition across studies (Armour et al., 2019). We analyzed the sham-irradiated control mice from each study to determine if such study effects could impact our comparison of results between the two investigations. If no study effects exist, we would expect these sham controls to manifest effectively indistinguishable compositions between the two studies. However, we instead find through a PERMANOVA analysis of the Bray–Curtis dissimilarity that there exist substantial and significant differences in the composition of the sham-irradiated control mouse microbiomes between the two investigations (*R*^2^ = 0.23387, *p* = 0.0002). These results suggest that study effects help drive the variation in results between these two studies. The effects could include, for example, differences in other types of environmental stress during transit or exposure to different microbes during transit or sham-irradiation or radiation exposure, although the housing, feeding, and handling were carried out in the same colonies and under the same general conditions for both studies. Regardless, the collective results of these two investigations suggest that sequential mixed particle beam radiation robustly elicits alterations to the composition of the mouse gut microbiome, but that sex is an important variable to consider when defining precisely how the microbiome responds to such exposures.

This study is among the earliest to demonstrate behavioral and cognitive effects of exposure to an increasingly complex charged particle radiation field. Astronauts will be exposed to the full spectrum of primary ions in the GCR along with secondary particles (including neutrons) during extended space flight outside the Earth’s magnetic field, including a Mars mission (Zeitlin et al., 2013; Hassler et al., 2014). The results in the present study suggest that both sex-independent effects of complex charged particle exposures and sex-dependent effects of such exposures are possible in astronauts. Furthermore, the observed interactions between radiation-associated changes in the gut microbiome and behavioral and cognitive performance are an area ripe for further investigation and potential intervention during long duration space flight.

## Data Availability Statement

The datasets presented in this study can be found in online repositories. The names of the repository/repositories and accession number(s) can be found in the article/Supplementary Material.

## Ethics Statement

The animal study was reviewed and approved by IACUC OHSU.

## Author Contributions

JR, AK, and MT conceived and designed the work. AF, JL, ET, and TS performed the experiments. JR, AF, JL, ET, SB, AH, KK, and TS analyzed the data. JR, AK, AF, TS, and MT wrote the manuscript. All coauthors contributed to the editing of the manuscript.

## Conflict of Interest

The authors declare that the research was conducted in the absence of any commercial or financial relationships that could be construed as a potential conflict of interest.

## Supplementary Material

FIGURE S1 Home cage of female **(A)** and male **(B)** mice that were sham irradiated or received six sequential beams. There was no effect of irradiation on activity levels of females or males during the light and dark periods.

FIGURE S2 Time female **(A)** and male **(B)** mice spent exploring objects in the object recognition test. ^#^*p* = 0.091.

FIGURE S3 Depressive-like behavior of sham-irradiated and irradiated female **(A)** and male **(B)** mice in the forced swim test. There was no effect of radiation on depressive-like behaviors in female or male mice.

FIGURE S4 Fear learning of sham-irradiated and irradiated female **(A,C)** and male **(B,D)** during the tones **(A,B)** and during the ISIs **(C,D)** in the fear conditioning test. There was no effect of radiation on fear learning of female or male mice.

FIGURE S5 Contextual fear memory of sham-irradiated and irradiated female **(A)** and male **(B)** mice. There was no effect of radiation on contextual fear memory of female or male mice. There was also no effect of radiation during the cued fear memory test in either female **(C)** nor male **(D)** mice.

FIGURE S6 Hippocampal BDNF **(A)**, hippocampal CD68 **(B)**, cortical MAP-2 **(C)**, and hippocampal MAP-2 **(D)** levels in sham-irradiated and irradiated mice. There was no effect of radiation on hippocampal BDNF, CD68, and MAP-2 levels or cortical MAP-2 levels.
