# Supplementary figures and images for: Effects of Six Sequential Charged Particle Beams on Behavioral and Cognitive Performance in B6D2F1 Female and Male Mice
Source: Front Physiol. 2020 Aug 28;11:959. doi: 10.3389/fphys.2020.00959 (PMC7485338; doi:10.3389/fphys.2020.00959)

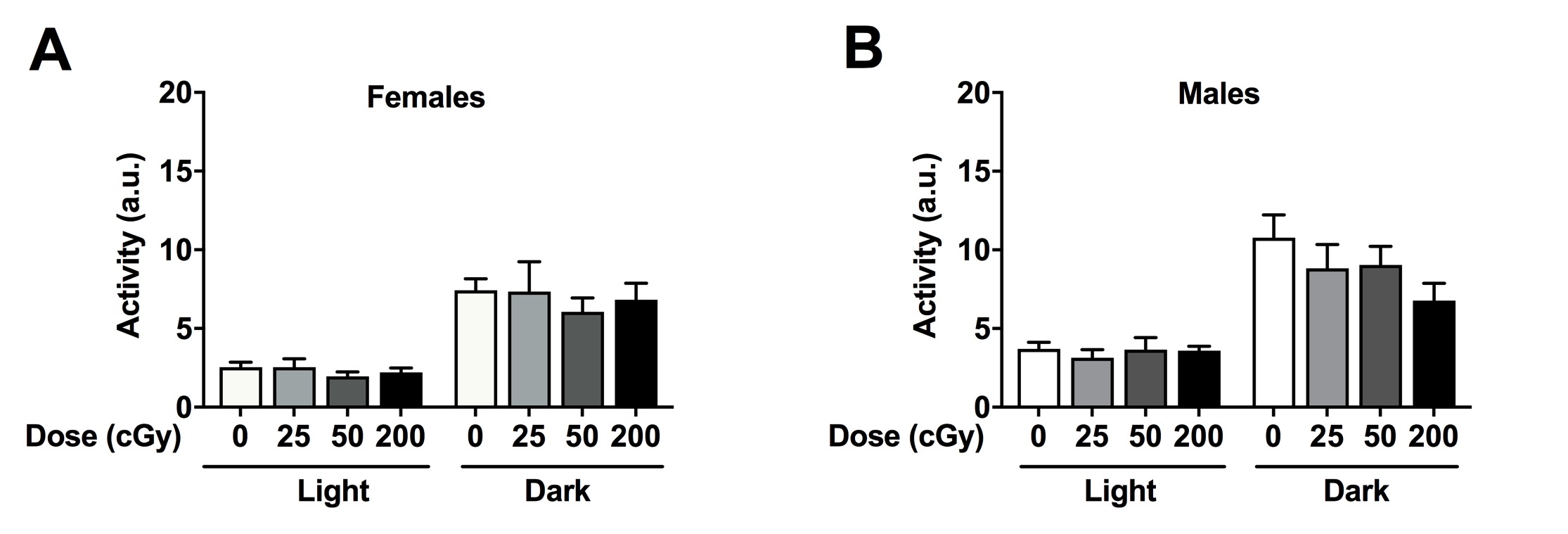

Supplement: FIGURE S1 — Home cage of female (A) and male (B) mice that were sham irradiated or received six sequential beams. There was no effect of irradiation on activity levels of females or males during the light and dark periods. [file Image_1.jpg]

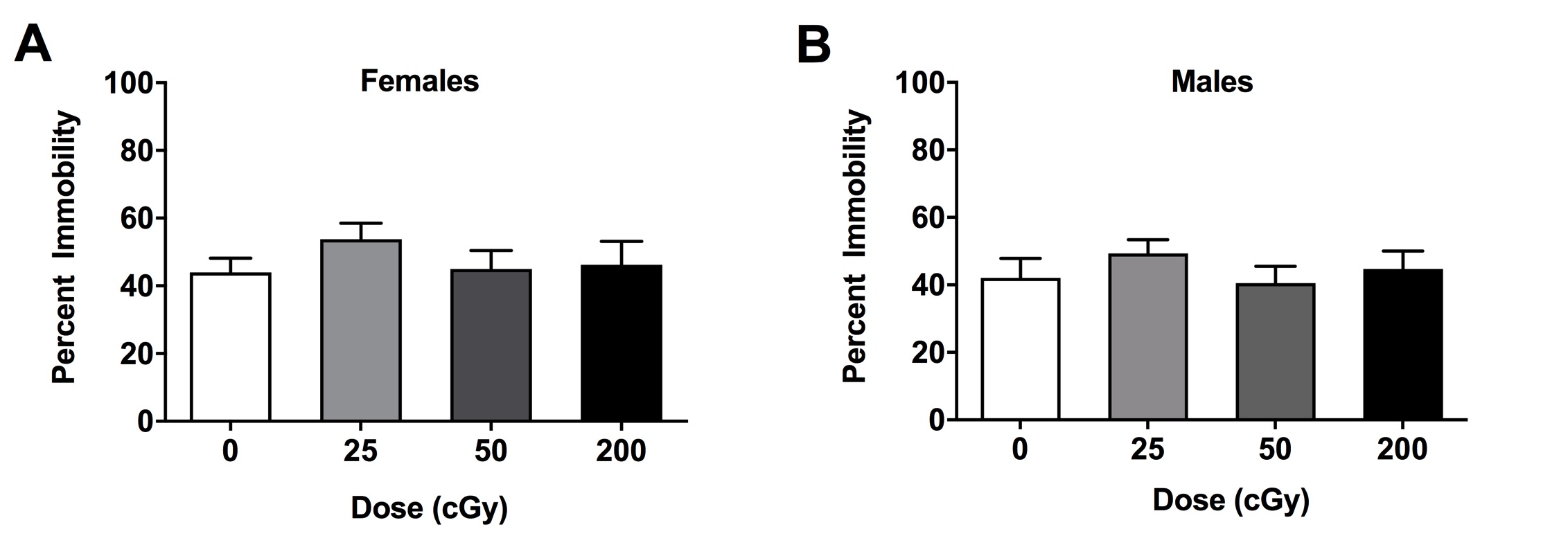

Supplement: FIGURE S2 — Time female (A) and male (B) mice spent exploring objects in the object recognition test. #p = 0.091. [file Image_2.jpg]

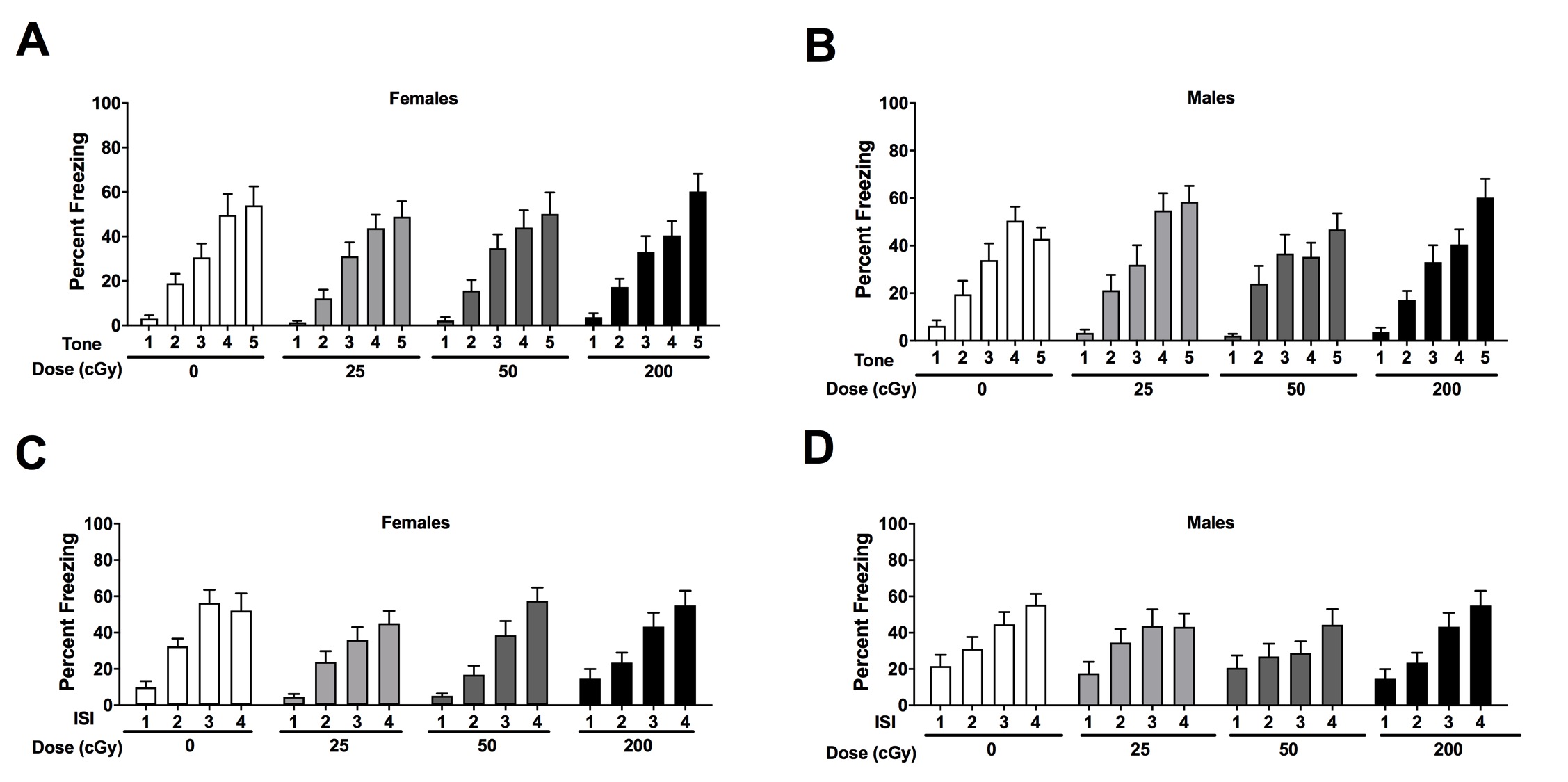

Supplement: FIGURE S3 — Depressive-like behavior of sham-irradiated and irradiated female (A) and male (B) mice in the forced swim test. There was no effect of radiation on depressive-like behaviors in female or male mice. [file Image_3.jpg]

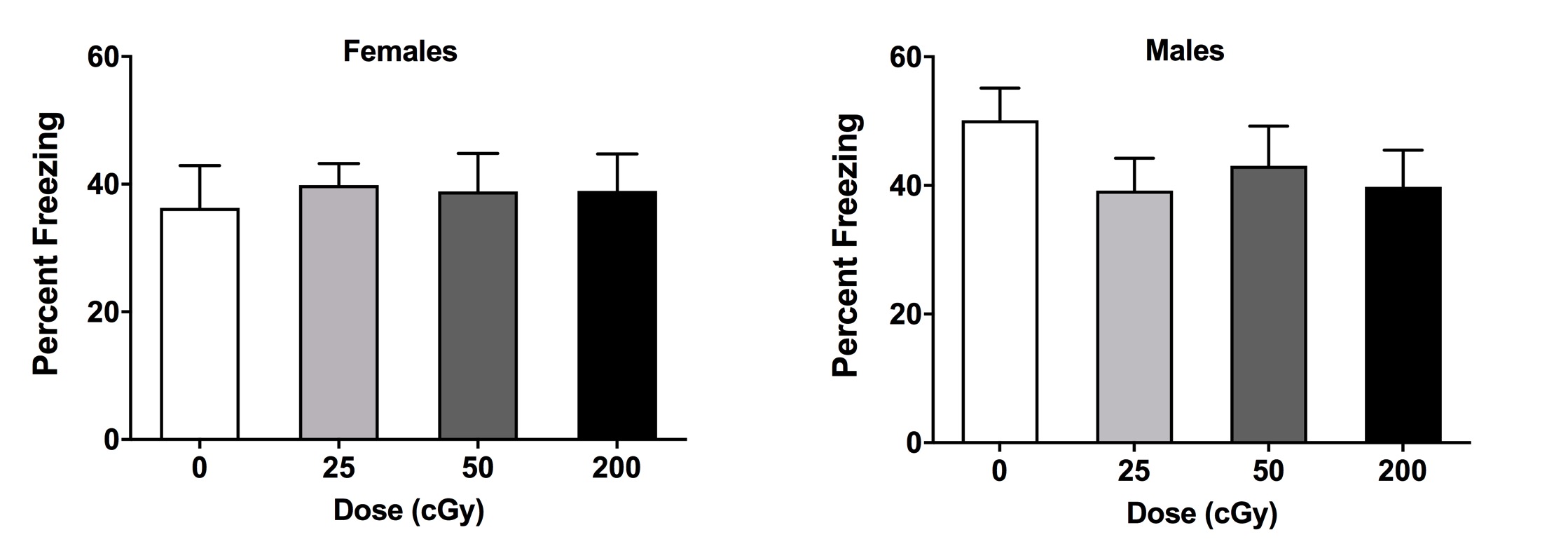

Supplement: FIGURE S4 — Fear learning of sham-irradiated and irradiated female (A,C) and male (B,D) during the tones (A,B) and during the ISIs (C,D) in the fear conditioning test. There was no effect of radiation on fear learning of female or male mice. [file Image_4.jpg]

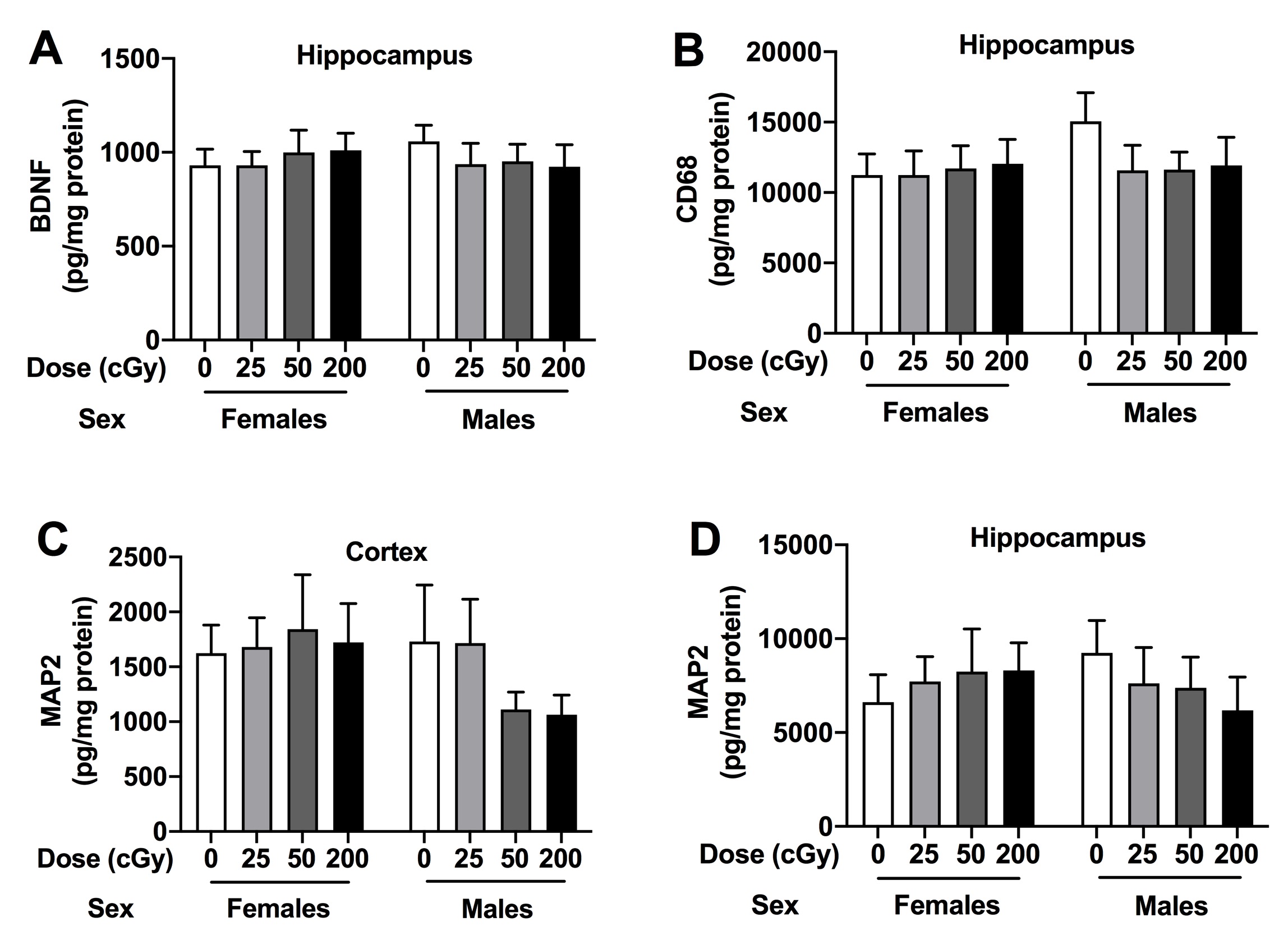

Supplement: FIGURE S5 — Contextual fear memory of sham-irradiated and irradiated female (A) and male (B) mice. There was no effect of radiation on contextual fear memory of female or male mice. There was also no effect of radiation during the cued fear memory test in either female (C) nor male (D) mice. [file Image_5.jpg]

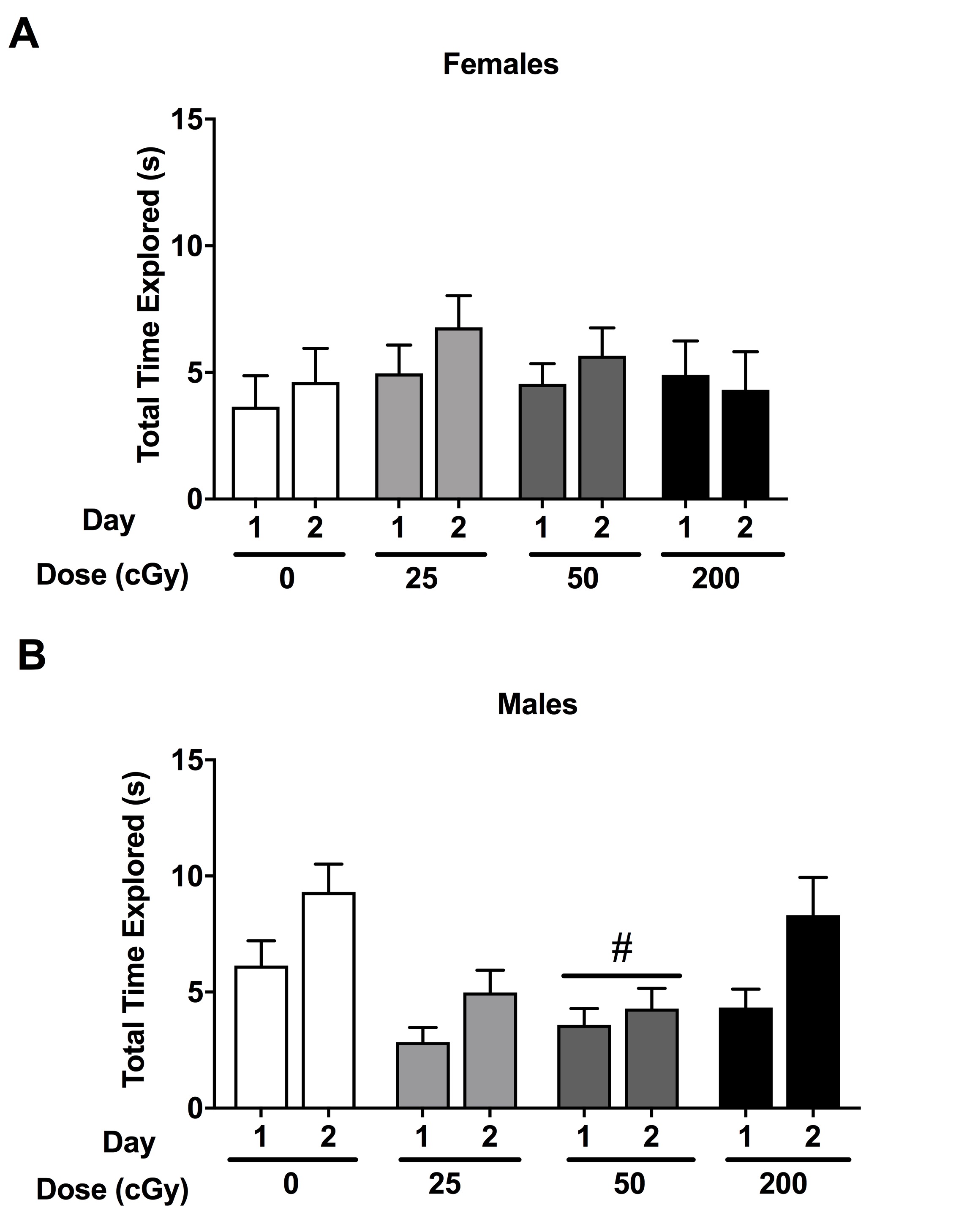

Supplement: FIGURE S6 — Hippocampal BDNF (A), hippocampal CD68 (B), cortical MAP-2 (C), and hippocampal MAP-2 (D) levels in sham-irradiated and irradiated mice. There was no effect of radiation on hippocampal BDNF, CD68, and MAP-2 levels or cortical MAP-2 levels. [file Image_6.JPEG]
